# Impact of smoke-free legislation on perinatal and infant mortality: a national quasi-experimental study

**DOI:** 10.1038/srep13020

**Published:** 2015-08-13

**Authors:** Jasper V Been, Daniel F Mackay, Christopher Millett, Jill P Pell, Onno CP van Schayck, Aziz Sheikh

**Affiliations:** 1 Division of Neonatology, Erasmus University Medical Centre – Sophia Children’s Hospital, PO Box 2060, 3000 CB Rotterdam, Netherlands; 2 Centre of Medical Informatics, Usher Institute of Population Health Sciences and Informatics, The University of Edinburgh, Edinburgh EH8 9AG, United Kingdom; 3 School for Public Health and Primary Care, Maastricht University, PO Box 616, 6200 MD Maastricht, Netherlands; 4 Department of Paediatrics, Maastricht University Medical Centre, PO Box 5800, 6202 AZ Maastricht, Netherlands; 5 Centre for Population and Health Sciences, University of Glasgow, Glasgow G12 8RZ, United Kingdom; 6 Department of Primary Care and Public Health, School of Public Health, Imperial College London, London W6 8RP, United Kingdom; 7 Division of General Internal Medicine and Primary Care, Brigham and Women’s Hospital, Boston MA 02120, USA; 8 Department of Medicine, Harvard Medical School, Boston MA 02115, USA

## Abstract

Smoke-free legislation is associated with improved early-life outcomes; however its impact on perinatal survival is unclear. We linked individual-level data with death certificates for all registered singletons births in England (1995–2011). We used interrupted time series logistic regression analysis to study changes in key adverse perinatal events following the July 2007 national, comprehensive smoke-free legislation. We studied 52,163 stillbirths and 10,238,950 live-births. Smoke-free legislation was associated with an immediate 7.8% (95%CI 3.5–11.8; p < 0.001) reduction in stillbirth, a 3.9% (95%CI 2.6–5.1; p < 0.001) reduction in low birth weight, and a 7.6% (95%CI 3.4–11.7; p = 0.001) reduction in neonatal mortality. No significant impact on SIDS was observed. Using a counterfactual scenario, we estimated that in the first four years following smoke-free legislation, 991 stillbirths, 5,470 cases of low birth weight, and 430 neonatal deaths were prevented. In conclusion, smoke-free legislation in England was associated with clinically important reductions in severe adverse perinatal outcomes.

Fifty years after the official recognition of its adverse health effects, tobacco smoking remains the primary cause of preventable mortality worldwide^1^. Besides affecting smokers’ health, exposure to second-hand smoke (SHS) is estimated to cause over 600,000 deaths annually worldwide^2^. Children account for over a quarter of these deaths and over half of the estimated 10.9 million disability-adjusted life years (DALYs) attributable to SHS exposure^2^. Among the key adverse outcomes associated with early-life SHS exposure are low birth weight (birth weight <2,500 grams)^1^,^3^, stillbirth^1^,^4^,^5^, and early-life mortality^1^, including sudden infant death syndrome (SIDS)^1^,^6^.

Population-level reductions in SHS exposure and active smoking can effectively be achieved by implementing legislation to prohibit smoking in enclosed public places and the workplace^7^. This is associated with significant health benefits among adults^7^,^8^. In a comprehensive assessment of its early-life health impact, we recently demonstrated that smoke-free legislation was associated with 10% reductions in both preterm birth and hospitalisations for childhood asthma^9^. The improvements in perinatal outcomes are likely mediated via reductions in maternal SHS exposure as well as active maternal smoking^9^,^10^,^11^,^12^,^13^,^14^. The data were however equivocal in relation to the impact on low birth weight babies^9^. We furthermore failed to identify any studies investigating the impact of smoke-free legislation on perinatal mortality, which was identified as a key knowledge gap^9^. Perinatal mortality is the primary contributor to the considerable global burden of under-five-mortality^15^. Given the urgent need for novel interventions in order to meet the fourth Millenium Development Goal of reducing this burden^16^, and the fact that only around 15% of the world’s population is currently covered by comprehensive smoke-free legislation^17^, the potential scope for improving early life outcomes is substantial.

We sought to address this by investigating the association between the 2007 national introduction of smoke-free legislation in England and various indicators of early-life mortality – namely stillbirth, neonatal mortality, and SIDS – in a national birth cohort of over 10 million singleton births. Given the conflicting evidence from previous studies^9^, we also evaluated the association between the legislation and low birth weight and updated our meta-analysis using these data. We hypothesised that smoke-free legislation would be associated with a reduced risk of developing the adverse perinatal outcomes evaluated.

## Results

### Study population

**Figure 1:**
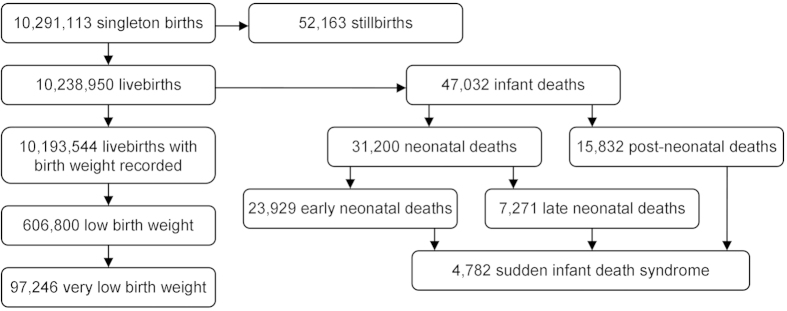
Flow diagram describing base population and number of individuals with primary and secondary outcomes .

During the study period, 10,291,113 singleton births were registered in England, including 52,163 stillbirths (0.5%; Fig. 1). Among the 10,238,950 singleton live-births, birth weight was recorded for 10,193,544 (99.6%), 606,800 (6.0%) of whom had low birth weight and 97,246 (1.0%) of whom had very low birth weight. Infant death occurred among 47,032 (0.5%) live-born infants and included 23,929 (0.2%) early neonatal deaths, 7,271 (0.1%) late neonatal deaths, and 15,832 (0.2%) post-neonatal deaths. 4,782 infant deaths were categorised as SIDS.

Stillbirth and early-life mortality were highly associated with low birth weight categories and low socioeconomic status, and less strongly with other demographic characteristics (Table 1). Important predictors of low birth weight included female sex, being a firstborn child, and low socioeconomic status (Table 2).

### Smoke-free legislation and early-life outcomes

Introduction of smoke-free legislation was associated with an immediate 7.8% (95% CI 3.5 to 11.8; p < 0.001) reduction in odds of being stillborn, a 3.9% (95% CI 2.6 to 5.1; p < 0.001) reduction in odds of having low birth weight, and a 7.6% (95% CI 3.4 to 11.7; p = 0.001) decrease in odds of neonatal mortality over-and-above the underlying temporal trend (Table 3 and Supplementary Table S1). No significant change in odds of SIDS was observed: 1.8% (95% CI −8.4 to 13.2; p = 0.74).

The reduction in neonatal mortality was primarily attributable to an impact on late neonatal deaths: −13.7% (95% CI −20.7 to −6.0; p = 0.001; Table 3 and Supplementary Table S2). A significant immediate reduction in overall odds of infant mortality was also observed: −6.3% (95% CI −9.6 to −2.9; p < 0.001). Smoke-free legislation was not associated with significant changes in the odds of the other secondary outcomes (Table 3).

### Sensitivity analyses

The findings were highly robust in pre-specified (Table 3) and post-hoc sensitivity analyses (Supplementary Table S3).

### Counterfactual estimates of cases averted

**Figure 2:**
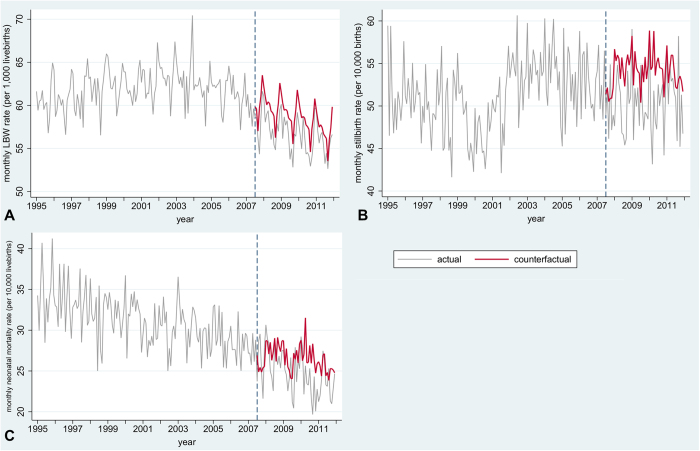
Actual and counterfactual rates for primary outcomes. Actual rates are based on all registered singleton births in England for stillbirth (n = 10,291,113) and all singleton live-births for the other outcomes (n = 10,238,950). Counterfactual rates are model-predicted rates based on a complete case scenario but without impact of smoke-free legislation (n = 9,984,278 for stillbirth, n = 9,933,349 for other outcomes). Only outcomes where smoke-free legislation had a significant impact are shown. A: low birth weight; B: stillbirth; C: neonatal mortality. Models are adjusted for non-linear underlying time trends, seasonality, maternal age, maternal marital status, sex, socioeconomic status, region, and urbanisation level and based on complete cases. Mortality models are also adjusted for birth weight. Dotted line represents introduction of smoke-free legislation. Note different scales on Y-axis.

**Figure 3:**
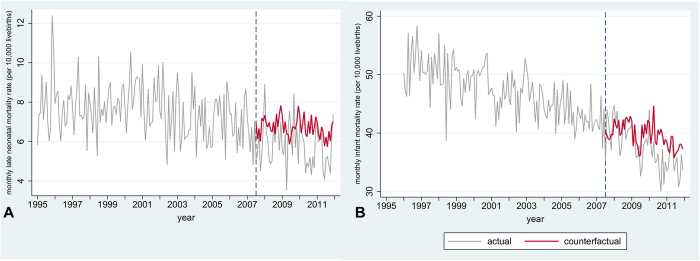
Actual and counterfactual rates for secondary outcomes. Actual rates are based on all registered live-births in England for infant mortality (n = 10,238,950) and on all babies alive at day seven after birth for late neonatal mortality (n = 10,207,750). Counterfactual rates are model-predicted rates based on a complete case scenario but without impact of smoke-free legislation (n = 9,911,272 for late neonatal mortality, n = 9,933,349 for infant mortality). Only outcomes where smoke-free legislation had a significant impact are shown. A: late neonatal mortality (n = 9,911,272); B: infant mortality (n = 9,933,349). Models are adjusted for non-linear time trends, month, maternal age, maternal marital status, sex, birth weight, socioeconomic status, region, and urbanisation level and based on complete cases. Dotted line represents introduction of smoke-free legislation. Note different scales on Y-axis.

Using a counterfactual scenario (Figs 2 and 3), we estimated that in the first four years following the implementation of smoke-free legislation in England 5,470 cases of low birth weight, 992 stillbirths, and 501 infant deaths, including 430 neonatal deaths, were averted.

## Discussion

Introduction of smoke-free legislation in England was associated with clinically important reductions in stillbirth, low birth weight, and neonatal and infant mortality.

Analysing over 10 million births this is, to the best of our knowledge, one of the largest studies to have investigated the impact of smoke-free legislation on early life health, and the first to focus on perinatal mortality^9^. We obtained data from national birth and death registries, which constitute the primary source for the production of English national statistics, thereby minimising risks of individuals being missed. Registration of individual data items attained a high level of completeness, with over 97% of individuals having complete data on all covariates included in the primary analyses.

Our study has a number of potential limitations. We had no individual-level information on maternal smoking status during pregnancy. We were therefore unable to assess whether a possible reduction in maternal smoking following introduction of smoke-free legislation, as observed by others, contributed to the observed improvement in perinatal outcomes^10^,^11^,^12^,^13^,^14^. Also, a gestational age indicator was missing from the data, as this information was only recorded as an individual item by the Office for National Statistics from 2006 onwards. It was thus not possible to distinguish between the most common underlying causes of low birth weight: intrauterine growth restriction (i.e. being small for gestational age) and reduced length of gestation (i.e. being born preterm)^18^. In a recent meta-analysis, we demonstrated that smoke-free legislation was associated with reductions in the incidence of preterm birth and of being very small for gestational age^9^, suggesting that both mechanisms are likely to have contributed to the observed reduction in low birth weight identified. Similarly, we were unable to assess whether the decreases in neonatal and infant mortality identified may in part have been mediated via a reduction in preterm births^9^. It is however important to note that although missing data on gestational age and maternal smoking status limited the opportunity for a detailed assessment of possible causal pathways (Supplementary Figure S1), this had no bearing on the validity of our findings in relation to the main hypothesis under investigation.

When interpreting our findings it should be noted that over 50% of employed adults already worked in a smoke-free workplace before the legislation was implemented^19^. There is thus a risk that our study under-estimated the true potential impact of smoke-free legislation, which may be larger in countries with a lower proportion of smoke-free environments prior to implementation. In this respect, it is also important to note that this study was undertaken in a country with comparatively good early life outcomes when judged against international standards^20^,^21^.

National public health interventions typically do not allow evaluation through randomised controlled study designs^22^. We therefore evaluated the impact of smoke-free legislation by undertaking a quasi-experimental study, accepting consequential limitations in causal inference^22^. It is in such contexts important to consider other factors that may help inform causal reasoning. Particularly noteworthy is that the reductions in adverse early-life outcomes we identified are in line with previous studies demonstrating particular perinatal health benefits as described above (i.e. reductions in low birth weight, being small for gestational age, and preterm birth)^11^,^12^,^13^,^23^,^24^,^25^,^26^,^27^,^28^. Furthermore, the link between smoke-free legislation and these reductions is highly plausible given that both SHS exposure and active smoking are well-established risk factors for adverse pregnancy outcomes and infant death^1^. Meta-analyses of studies among non-smoking women have shown that SHS exposure during pregnancy was associated with a 1.32 (95% CI 1.07 to 1.63; p = 0.02) times increased risk of low birth weight and a 1.23 (95% CI 1.09 to 1.38; p < 0.001) times increased risk of stillbirth^3^,^4^. Furthermore, a dose-dependent inverse relationship between maternal urinary cotinine levels (as a proxy for SHS exposure) and offspring birth weight has recently been described^29^. Contemporary studies assessing the impact of SHS exposure on neonatal mortality are however lacking^4^,^30^. As for active maternal smoking during pregnancy, a reduction in which is highly likely to be on the causal pathway between smoke-free legislation and improved perinatal outcomes (Supplementary Figure S1)^1^,^10^,^11^,^12^,^13^,^14^ there is a strong association with adverse pregnancy outcomes as demonstrated by studies reporting a 36–60% increased risk for stillbirth and a 20% increased risk for neonatal mortality^1^,^5^. Observational and experimental studies have found that maternal smoking cessation normalises these risks^1^,^31^,^32^,^33^. For example, a randomised controlled trial of a counselling intervention that successfully reduced SHS exposure during pregnancy reported a significant improvement in birth outcomes^34^. We are unaware of other public health interventions or changes in perinatal practice co-occurring with the implementation of smoke-free legislation in England that may have been responsible for such substantial immediate reductions in several key adverse perinatal outcomes.

Although no previous studies have investigated the impact of smoke-free legislation on perinatal mortality, several have studied its effect on birth weight^11^,^12^,^13^,^23^,^24^,^25^,^26^,^35^. In a recent meta-analysis of six studies, no significant overall impact on low birth weight could be demonstrated: −1.7% (95% CI −5.1 to 1.6; p = 0.31)^9^. We identified no new eligible studies in an update of this systematic review and meta-analysis (Supplementary Figure S2), focusing on (very) low birth weight and early-life mortality and following the methods described earlier^36^ Adding data from the current study to the existing meta-analyses, the overall reductions in the risk of low birth weight (7 studies, >12.1 million subjects: −2.20% [95% CI −4.95 to 0.54], p = 0.115) and very low birth weight (3 studies, >10.2 million subjects: −2.61% [95% CI −12.15 to 6.95], p = 0.591) following introduction of smoke-free legislation were not statistically significant. Of note however, the two studies that demonstrated a significant reduction in low birth weight were performed in countries where smoke-free legislation has been particularly comprehensive and compliance high^12^. Meta-analyses of smoke-free legislation and adult health have consistently shown that its health impact is larger when legislation is more comprehensive^8^,^37^. Additional studies are needed to study whether the same accounts for perinatal outcomes, which could further strengthen the case for WHO recommendations to implement comprehensive smoke-free laws^17^.

The association between smoke-free legislation and mortality was primarily attributable to a reduction in late neonatal mortality, which likely relates to the recognised association between antenatal smoke exposure and common causes of death in the late neonatal period^38^ such as necrotising enterocolitis^39^, bronchopulmonary dysplasia^40^,^41^, and sepsis^42^. We were unable to assess the differential association between smoke-free legislation and specific causes of death (except for SIDS) as this information was lacking from our data. Post-hoc sensitivity analyses indicate that the reduction in early-life mortality following smoke-free legislation was likely related to mechanisms other than improvement (i.e. increase) in birth weight.

Whereas we identified an overall association between smoke-free legislation and reduced infant mortality, we were surprised by the finding that SIDS was not affected^1^,^5^. Boldo *et al.* previously estimated that SHS exposure was responsible for 310–420 SIDS cases annually in Europe, amounting to 1.6 excess cases per 100,000 in 2005^6^. It is possible that our study lacked power to detect small changes in this rare outcome although the point estimate was not suggestive of possible benefit (Table 3). As SIDS is a diagnosis *per exclusionem*, temporal changes in the diagnostic approach towards unexplained infant death (e.g. changes in the proportion of explained deaths, changes in *post-mortem* examination rates, inter-observer variation in diagnostic criteria) may have resulted in unexplained variation^43^,^44^. Misclassification of SIDS may furthermore have influenced the data^45^. Of note, a recent multi-country ecological analysis found that higher tobacco taxes, but not smoke-free laws, were associated with significant reductions in SIDS rates^46^.

The impact of smoke-free legislation on perinatal and early-life health is likely to have been mediated via several routes. A number of previous studies have demonstrated important drops in maternal smoking during pregnancy following implementation of smoke-free legislation^1^,^10^,^11^,^12^,^13^,^14^. In Scotland, for example, maternal smoking during pregnancy dropped from 25.4% to 18.8%^12^. At the same time, similar reductions in low birth weight were identified among women who smoked and those who did not smoke during pregnancy^12^, suggesting that at least part of the effect is mediated via mechanisms other than reducing active maternal smoking. Improvements in perinatal outcomes were observed among Norwegian mothers whose workplace became smoke-free^13^. In Belgium, both the smoking ban in pubs and restaurants, as well as the workplace ban benefitted perinatal health^23^. Smoke-free legislation has furthermore been associated with important drops in smoking in the home through social norm spreading^47^,^48^,^49^,^50^. The impact observed in our study is therefore likely to result from a mixture of reduced active smoking and reduced SHS exposure in the workplace, public places, and the home environment.

Our findings add an important new dimension to the emerging evidence on the benefits of smoke-free legislation to child health^9^,^51^, adding to the already well-established broad range of health benefits among adults^7^,^8^. Smoke-free laws are an inexpensive and efficient means to achieve sizeable improvements in population health^52^. Such laws are supported by the public, with support increasing further (particularly amongst smokers) following implementation^53^. Considering that only around 15% of the world’s population is currently protected by comprehensive smoke-free laws^17^, and that low birth weight and perinatal mortality remain the primary causes of childhood morbidity and mortality worldwide^18^,^21^,^54^, accelerated action to implement smoke-free legislation is likely to help save considerable numbers of young lives across the globe and through doing so enhance the much-needed progress in meeting the fourth Millennium Development Goal.

Since the majority of the burden of early-life morbidity and mortality occurs in low- and middle-income countries^18^,^21^, there is a particular need for studies assessing the impact of smoke-free legislation in these regions^9^. Work is also needed to determine the impact of tobacco control policies on marginalised populations in high-income country settings, as variation in maternal smoking across socioeconomic subgroups has been shown to account for approximately one-third of the inequalities in stillbirths and infant deaths^55^. Finally, research also needs to investigate the differential early-life health impact of varying degrees of comprehensiveness of smoke-free laws (e.g. extending the legislation to outdoor public places, private cars and homes) and different approaches to enforcement and success with compliance^8^.

In conclusion, we present evidence that implementation of smoke-free legislation in England was associated with substantial perinatal and early-life benefits, with over 5,000 cases of low birth weight and almost 1,500 deaths averted within four years.

## Methods

This study was performed according to a pre-specified and registered research protocol (ClinicalTrials.gov NCT02039583). Using a national dataset of all singleton births registered in England between 1995 and 2011, we analysed the association between the July 2007 implementation of smoke-free legislation and the odds of stillbirth, low birth weight, and early-life mortality.

### Ethical considerations

This study was reviewed by the National Health Services (NHS) South East Scotland Research Ethics Service and The University of Edinburgh’s Centre for Population Health Sciences Ethics Review Group. Both committees provided an exemption from formal ethical assessment based on the use of anonymised, unidentifiable data.

### Implementation of smoke-free legislation

The intervention under study was the implementation of smoke-free legislation in England on 1 July 2007^56^. From this date, smoking was prohibited in enclosed public places and workplaces in England, with very few exemptions (e.g. specialist tobacconist shops, designated rooms in palliative care hospices and in prisons). Immediate and sustained high levels of compliance with the smoke-free law have been attained, with over 98% of public premises and vehicles (e.g. taxis, private hire vehicles, coaches, and buses) found to be smoke-free in the first year following its implementation^57^.

### Outcome definitions

Four primary outcomes were evaluated: low birth weight (live-birth with birth weight <2,500 grams); stillbirth (intrauterine death from 24 weeks of gestation); neonatal death (death in the first 28 days of life); SIDS (death within the first year of life coded on the death certificate as International Classification of Diseases (ICD) 10-U R95, or R99 with no other specification).

In addition, we considered the following secondary outcomes: very low birth weight (live-birth with birth weight <1,500 grams); early neonatal death (death in the first week of life); late neonatal death (death between 7 and 28 days of life); post-neonatal death (death between 28 days of life and the first birthday); and infant death (death within the first year of life).

Data on gestational age were not available through the Office for National Statistics before 2006, restricting the pre-legislation period to only 1.5 years. Given the recognised seasonal variation in gestational age we considered this period insufficient to estimate underlying trends pre-legislation, and therefore decided *a priori* not to involve preterm birth as an outcome or intermediate variable in our analyses.

### Data sources and study population

Individual-level data on all registered singleton births in England between 1 January 1995 and 31 December 2011 were obtained via the government’s Office for National Statistics. Data were linked by the Office for National Statistics to death certificates for stillbirths and for all infants dying before their first birthday. To minimise the risks of individuals being identified, individual-level continuous variables were categorized as follows: month and year of birth; month and year of death; timing of death (stillbirth/early neonatal/late neonatal/post-neonatal); sex; birth weight (<1,000 grams/1,000–1,499 grams/1,500–2,499 grams/2,500–3,999 grams/≥4,000 grams); maternal marital status (married /not married); maternal parity; and maternal age (<20 years/20–24 years/25–29 years/30–34 years/35–39 years/≥40 years). The individual-level data also included Government Office Region, urbanisation level (urban/rural, based on Office for National Statistics classification for small area geographies), and socioeconomic status measured using the Index of Multiple Deprivation (in quintiles), these being derived from maternal post code of residence. The Index of Multiple Deprivation is a relative measure of deprivation at the small-area level^58^; an aggregate score is produced from 38 indicators in seven domains, namely: income; employment; health; education; crime; access to services; and living environment^58^.

### Statistical analyses

The patterning of demographic data was initially tabulated by timing of death and birth weight categories. Separate logistic regression models using individual-level data were then developed to investigate the association between the introduction of smoke-free legislation and the odds of developing each outcome^12^. Akaike’s and Schwarz’s Bayesian information criteria (AIC and BIC, respectively) were used to select the optimal model from among six options (Supplementary Figure S3): a linear time-trend model with a sudden (‘step’) change in the odds of developing the outcome at the time of introduction of smoke-free legislation; a linear time-trend model with a gradual (‘slope’) change in odds following the introduction of smoke-free legislation; a linear time-trend model with both a step and a slope change; and three models with a step change only and the underlying time trends being modelled via linear, quadratic, and cubic B-splines, respectively^59^. A continuous time variable based on month of birth was used for linear time trend models. Via addition of these temporal trend terms all models thus accounted for the pre-existing (i.e. before introduction of smoke-free legislation) underlying trend in the odds of developing each outcome. The step change was modeled using a dummy variable code ‘0’ before and ‘1’ after the introduction of smoke-free legislation^19^. An interaction term between this dummy and the continuous time variable was included to model a slope change^51^,^60^. In addition, a categorical variable for month was added to each model to account for any seasonality in the data^51^,^60^.

The following categorical covariates were included in each model to account for potential individual-level confounding: sex; maternal age; maternal marital status; Index of Multiple Deprivation quintile; region; and urbanisation level. All mortality-related outcomes were also adjusted for birth weight.

Among the model covariates, data on parity were missing for a substantial portion of the study population (41.3%), as this variable had been recorded for married women only. All other covariates had 0–3% missing data. Since logistic regression analysis needs to be undertaken on cases with complete data for all covariates, parity was excluded from the primary models to maximise power. We performed two sets of pre-specified sensitivity analyses to investigate potential residual confounding resulting from exclusion of parity as a covariate. In the first set, models were re-run with parity included, analysing individuals with complete data only. In the second set, multiple imputations were performed using chained equations to impute missing data for parity, creating five unique datasets^12^. Models were then re-run on the imputed data. Sensitivity analyses were conducted for the primary outcomes only to minimise risks associated with multiple testing.

A change in odds of developing low birth weight following the introduction of smoke-free legislation is potentially on the causal pathway between smoke-free legislation and any observed impact on early-life mortality (Supplementary Figure S1). We therefore performed additional post-hoc sensitivity analyses to test whether exclusion of birth weight as a covariate from the models had any impact on the observed association between smoke-free legislation and early-life mortality.

In order to estimate the absolute impact of smoke-free legislation for outcomes that were significantly affected, we developed counterfactual scenarios^51^,^60^. For each individual, a predicted risk of developing the outcome was calculated using the Betas from the primary models, but setting the ban dummy at ‘0’ for the entire study period. This counterfactual risk thus represented the theoretical risk of developing the outcome, had smoke-free legislation not been implemented. We then subtracted the actual risk from the counterfactual risk, producing an excess risk for each individual. These were summed for the first four full years following smoke-free legislation, producing an estimate of the total number of cases averted for each outcome.

All analyses were undertaken using Stata SE version 12.0 (Statacorp, TX).

### Sample size considerations

As we used the maximum time span and population available, sample size calculations were redundant. Given the national nature of this evaluation, we estimated that we would have adequate power to detect clinically relevant temporal changes in the outcomes of interest^9^. A number of studies have previously assessed the impact of smoke-free legislation on low birth weight^11^,^12^,^13^,^23^,^24^,^25^,^26^. Our approach was comparable to that previously employed in Scotland^12^. Using data on 757,795 deliveries occurring between 1996 and 2009, this work showed an immediate −9.9% (95% CI −14.2 to −5.2; p < 0.001) drop in low birth weight babies^12^. Given the longer study period (1995–2011) and the much larger population size (n > 10 million), the current study was expected to have sufficient power to detect a similar reduction in low birth weight babies in England, if present. Earlier studies of the impact of smoke-free laws on SIDS are hampered by their ecological design and these were therefore not suitable to inform deliberations on power considerations for the current study^27^,^46^.

## Additional Information

**How to cite this article**: Been, J. V. *et al.* Impact of smoke-free legislation on perinatal and infant mortality: a national quasi-experimental study. *Sci. Rep.* **5**, 13020; doi: 10.1038/srep13020 (2015).

## Supplementary Material

Supplementary Information

## Figures and Tables

**Table 1:** Demographic characteristics according to mortality categories.

	**Live-births (n = 10,238,950)**	**Stillbirths (n = 52,163)**	**Infant deaths (n = 47,032)**					
			**All infant deaths (n = 47,032)**	**Neonatal deaths (n = 31,200)**			**Post-neonatal deaths (n = 15,832)**	**SIDS (4,782)**
				**All neonatal deaths (n = 31,200)**	**Early neonatal deaths (n = 23,929)**	**Late neonatal deaths (n = 7,271)**		
Maternal age (years)								
<20	691,251 (6.8)	4,229 (8.1)	4,999 (10.6)	2,886 (9.3)	2,132 (8.9)	754 (10.4)	2,113 (13.3)	953 (19.9)
20–24	1,926,659 (18.8)	10,007 (19.3)	10,356 (22.0)	6,457 (20.7)	4,884 (20.4)	1,573 (21.6)	3,899 (24.6)	1,465 (30.6)
25–29	2,874,147 (28.1)	13,689 (26.2)	12,392 (26.3)	8,354 (26.8)	6,392 (26.7)	1,962 (27.0)	4,038 (25.5)	1,077 (22.5)
30–34	2,954,400 (28.9)	13,668 (26.2)	11,225 (23.9)	7,848 (25.2)	6,131 (25.6)	1,717 (23.6)	3,377 (21.3)	793 (16.6)
35–39	1,487,027 (14.5)	8,046 (15.4)	6,330 (13.5)	4,461 (14.3)	3,476 (14.5)	985 (13.5)	1,869 (11.8)	403 (8.4)
≥40	305,415 (3.0)	2,454 (4.7)	1,730 (3.7)	1,194 (3.8)	914 (3.8)	280 (3.9)	536 (3.4)	91 (1.9)
Missing	51 (0.0)	0 (0.0)	0 (0.0)	0 (0.0)	0 (0.0)	0 (0.0)	0 (0.0)	0 (0.0)
Parity								
0	2,441,463 (40.6)	10,849 (38.0)	10,353 (42.6)	7,637 (45.7)	5,950 (46.1)	1,687 (44.3)	2,716 (35.7)	386 (27.4)
1	2,188,681 (36.4)	9,040 (31.6)	7,141 (29.4)	4,748 (28.4)	3,643 (28.3)	1,105 (29.0)	2,393 (31.4)	451 (32.0)
2	884,312 (14.7)	4,630 (16.2)	3,712 (15.3)	2,349 (15.2)	1,788 (13.9)	561 (14.7)	1,363 (17.9)	302 (21.4)
≥3	496,592 (8.3)	4,053 (14.2)	3,102 (12.8)	1,960 (11.7)	1,507 (11.7)	453 (11.9)	1,142 (15.0)	270 (19.2)
Missing	4,227,902 (41.3)	23,591 (45.2)	22,724 (48.3)	14,093 (46.5)	11,041 (46.1)	3,465 (47.7)	8,218 (51.9)	3,373 (70.5)
Marital status								
Married	6,011,122 (58.7)	28,573 (54.8)	24,308 (51.7)	16,694 (53.5)	12,888 (53.9)	3,806 (52.3)	7,614 (48.1)	1,409 (29.5)
Not married	4,227,828 (41.3)	23,590 (45.2)	22,724 (48.3)	14,506 (46.5)	11,041 (46.1)	3,465 (47.7)	8,218 (51.9)	3,373 (70.5)
Missing	0 (0.0)	0 (0.0)	0 (0.0)	0 (0.0)	0 (0.0)	0 (0.0)	0 (0.0)	0 (0.0)
Sex								
Male	5,251,746 (51.3)	27,509 (52.7)	26,571 (56.5)	17,532 (56.3)	13,479 (56.3)	4,083 (56.2)	9,009 (56.9)	2,821 (59.0)
Female	4,987,201 (48.7)	24,654 (47.3)	20,461 (43.5)	13,638 (43.7)	10,450 (43.7)	3,188 (43.8)	6,823 (43.1)	1,961 (41.0)
Missing	3 (0.0)	0 (0.0)	0 (0.0)	0 (0.0)	0 (0.0)	0 (0.0)	0 (0.0)	0 (0.0)
Birth weight (grams)								
<1000	41,737 (0.4)	15,719 (30.4)	16,073 (35.3)	13,826 (46.3)	11,496 (50.6)	2,330 (32.8)	2,247 (14.3)	66 (1.4)
1000–1499	55,509 (0.5)	6,500 (12.6)	3,708 (8.1)	2,732 (9.2)	2,011 (8.8)	721 (10.2)	976 (6.2)	151 (3.2)
1500–2499	509,554 (5.0)	11,722 (22.7)	7,175 (15.8)	4,202 (14.1)	3,055 (13.4)	1,147 (16.2)	2,973 (19.0)	869 (18.3)
2500–3999	8,422,413 (82.6)	15,719 (30.4)	17,009 (37.4)	8,174 (27.4)	5,482 (24.1)	2,692 (37.9)	8,835 (56.4)	3442 (74.5)
≥4000	1,164,331 (11.4)	2,051 (4.0)	1,538 (3.4)	906 (3.0)	694 (3.1)	212 (3.0)	632 (4.0)	220 (4.6)
Missing	45,406 (0.4)	453 (0.9)	1,529 (3.3)	1,360 (4.4)	1,191 (5.0)	169 (2.3)	169 (1.1)	34 (0.7)
IMD quintile								
1 (most deprived)	1,817,749 (18.2)	11,127 (21.7)	10,359 (22.6)	6,910 (22.8)	5,296 (22.8)	1,614 (22.6)	3,449 (22.2)	991 (21.4)
2	2,618,898 (26.3)	14,467 (28.2)	13,546 (29.6)	8,980 (29.6)	6,895 (29.7)	2,085 (29.2)	4,566 (29.4)	1,253 (27.0)
3	2,241,333 (22.5)	11,099 (21.6)	9,954 (21.7)	6,598 (21.7)	5,005 (21.6)	1,593 (22.3)	3,356 (21.6)	1,079 (23.3)
4	1,953,029 (19.6)	8,984 (17.5)	7,536 (16.4)	4,892 (16.1)	3,737 (16.1)	1,155 (16.2)	2,644 (17.0)	816 (17.6)
5 (least deprived)	1,344,295 (13.5)	5,664 (11.0)	4,466 (9.7)	2,960 (9.8)	2,264 (9.8)	696 (9.7)	1,506 (9.7)	497 (10.7)
Missing	263,646 (2.6)	822 (1.6)	1,171 (2.5)	860 (2.8)	732 (3.1)	128 (1.8)	311 (2.0)	146 (3.1)
Region								
Greater London	1,891,740 (19.0)	11,258 (21.9)	9,163 (20.0)	6,046 (19.9)	4,551 (19.6)	1,495 (20.9)	3,117 (20.1)	716 (15.4)
North East	471,619 (4.7)	2,458 (4.8)	2,102 (4.6)	1,347 (4.4)	998 (4.3)	349 (4.9)	755 (4.9)	277 (6.0)
North West	1,355,390 (13.6)	7,131 (13.9)	6,907 (15.1)	4,439 (14.6)	3,360 (14.5)	1,079 (15.1)	2,468 (15.9)	879 (18.8)
Yorkshire and the Humber	1,000,678 (10.0)	5,517 (10.7)	5,286 (11.5)	3,421 (11.3)	2,568 (11.1)	854 (12.0)	1,865 (12.0)	493 (10.6)
East Midlands	726,852 (7.3)	3,615 (7.0)	3,354 (7.3)	2,316 (7.6)	1,775 (7.6)	531 (7.4)	1,074 (6.9)	348 (7.5)
West Midlands	1,097,732 (11.0)	5,924 (11.5)	6,292 (13.7)	4,411 (14.5)	3,557 (15.3)	853 (11.9)	1,881 (12.1)	539 (11.6)
East of England	1,026,552 (10.3)	4,670 (9.1)	3,819 (8.3)	2,494 (8.2)	1,931 (8.3)	563 (7.9)	1,325 (8.5)	364 (7.9)
South East	812,769 (8.1)	3,508 (6.8)	2,650 (5.8)	1,724 (5.7)	1,317 (5.7)	407 (5.7)	926 (6.0)	326 (7.0)
South Central	721,568 (7.2)	3,422 (6.7)	2,833 (6.2)	1,862 (6.1)	1,390 (6.0)	472 (6.6)	971 (6.3)	281 (6.1)
South West	870,404 (8.7)	3,838 (7.5)	3,455 (7.5)	2,280 (7.5)	1,749 (7.7)	541 (7.6)	1,139 (7.3)	420 (9.1)
Missing	263,646 (2.6)	822 (1.6)	1,171 (2.5)	860 (2.8)	732 (3.1)	128 (1.8)	311 (2.0)	146 (3.1)
Urbanisation level								
Urban	9,759,136 (97.8)	50,371 (98.1)	45,115 (98.4)	29,828 (98.3)	22,787 (98.2)	7,041 (98.6)	15,287 (98.5)	4,563 (98.4)
Rural	216,168 (2.2)	970 (1.9)	746 (1.6)	512 (1.7)	410 (1.8)	102 (1.4)	234 (1.5)	73 (1.6)
Missing	263,646 (2.6)	822 (1.6)	1,171 (2.5)	860 (2.8)	732 (3.1)	128 (1.8)	311 (2.0)	146 (3.1)

Numbers represent numbers of babies in each stratum. Percentages in parentheses relate to all babies with valid data for that particular variable. For missing data, percentages of the full sample are given. SIDS = sudden infant death syndrome; IMD = Index of Multiple Deprivation.

**Table 2:** Demographic characteristics according to birth weight categories.

	**Birth weight ≥ 2,500 g (n = 9,586,744)**	**Low birth weight (n = 606,800)**	**Very low birth weight (n = 97,246)**	**Birth weight missing (n = 45,406)**
Maternal age (years)				
<20	632,058 (6.6)	56,372 (9.3)	8,999 (9.3)	2,821 (6.2)
20–24	1,785,158 (18.6)	133,010 (21.9)	19,669 (20.2)	8,491 (18.7)
25–29	2,697,989 (28.1)	163,814 (27.0)	25,485 (26.2)	12,344 (27.2)
30–34	2,791,293 (29.1)	150,470 (24.8)	24,480 (25.2)	12,637 (27.9)
35–39	1,397,224 (14.6)	82,428 (13.6)	14,705 (15.1)	7,375 (16.3)
≥40	283,019 (3.0)	20,706 (3.4)	3,908 (4.0)	1,691 (3.7)
Missing	4 (0.0)	0 (0.0)	0 (0.0)	47 (0.1)
Parity				
0	2,271,216 (40.0)	156,788 (50.1)	24,541 (50.2)	13,495 (52.6)
1	2,095,213 (36.9)	86,370 (27.6)	13,165 (26.9)	7,098 (27.7)
2	841,217 (14.8)	40,002 (12.8)	6,282 (12.9)	3,093 (12.1)
≥3	465,144 (8.2)	29,492 (9.4)	4,875 (10.0)	1,956 (7.6)
Missing	3,913,954 (40.8)	294,148 (48.5)	48,383 (49.8)	19,800 (43.6)
Marital status				
Married	5,672,849 (59.2)	312,659 (51.5)	48,865 (50.2)	25,614 (56.4)
Not married	3,913,895 (40.8)	294,141 (48.5)	48,381 (49.8)	19,792 (43.6)
Missing	0 (0.0)	0 (0.0)	0 (0.0)	0 (0)
Sex				
Male	4,939,679 (51.5)	288,753 (47.6)	49,784 (51.2)	23,314 (51.3)
Female	4,647,062 (48.5)	318,047 (52.4)	47,462 (48.8)	22,092 (48.7)
Missing	3 (0.0)	0 (0.0)	0 (0.0)	0 (0)
IMD quintile				
1 (most deprived)	1,676,895 (18.0)	129,769 (21.7)	21,871 (22.8)	11,085 (26.4)
2	2,439,576 (26.1)	168,089 (28.0)	27,599 (28.8)	11,233 (26.8)
3	2,100,475 (22.5)	132,485 (22.1)	21,227 (22.1)	8,373 (20.0)
4	1,842,889 (19.7)	102,493 (17.1)	15,510 (16.1)	7,647 (18.2)
5 (least deprived)	1,274,139 (13.7)	66,542 (11.0)	9,692 (10.1)	3,614 (8.6)
Missing	252,770 (2.6)	7,422 (1.2)	1,347 (1.4)	3,454 (7.6)
Region				
Greater London	1,754,925 (18.8)	120,352 (20.1)	21,381 (22.3)	16,463 (39.2)
North East	440,830 (4.7)	29,295 (4.9)	4,579 (4.8)	1,494 (3.6)
North West	1,265,206 (13.6)	85,422 (14.3)	13,108 (13.7)	4,762 (11.4)
Yorkshire and the Humber	932,580 (10.0)	64,903 (10.8)	9,851 (10.7)	3,195 (7.6)
East Midlands	680,606 (7.3)	45,086 (7.5)	6,932 (7.2)	1,160 (2.8)
West Midlands	1,020,237 (10.9)	75,470 (12.6)	12,132 (12.7)	2,025 (4.8)
East of England	969,400 (10.4)	54,056 (9.0)	8,422 (8.8)	3,096 (7.4)
South East	769,979 (8.2)	40,383 (6.7)	5,996 (6.3)	2,407 (5.7)
South Central	680,340 (7.3)	38,901 (6.5)	6,345 (6.6)	2,327 (5.5)
South West	819,871 (8.8)	45,510 (7.6)	7,153 (7.5)	5,023 (12.0)
Missing	252,770 (2.6)	7,422 (1.2)	1,347 (1.4)	3,454 (7.6)
Urbanisation level				
Urban	9,128,455 (97.4)	589,290 (98.3)	94,414 (98.6)	41,391 (98.7)
Rural	205,519 (2.6)	10,088 (1.7)	1,485 (1.4)	561 (1.3)
Missing	252,770 (2.6)	7,422 (1.2)	1,347 (1.4)	3,454 (7.6)

Numbers represent numbers of live-born babies in each stratum. Percentages in parentheses relate to all babies with valid data for that particular variable. For missing data, percentages of the full sample are given. IMD = Index of Multiple Deprivation.

**Table 3:** Impact of smoke-free legislation on primary and secondary outcomes.

					**Sensitivity analyses**							
	**Primary analysis**				**Model 1**				**Model 2**			
	**N**	**OR**	**95%CI**	**P-value**	**N**	**OR**	**95%CI**	**P-value**	**N**	**OR**	**95%CI**	**P-value**
Primary outcomes												
Low birth weight	9,933,349	0.961	0.949–0.974	<0.001	5,822,837	0.980	0.962–0.998	0.032	9,933,349	0.963	0.951–0.976	<0.001
Stillbirth	9,984,278	0.922	0.881–0.965	<0.001	5,850,909	0.910	0.855–0.970	0.003	9,984,278	0.919	0.878–0.962	<0.001
Neonatal mortality	9,933,349	0.924	0.883–0.966	0.001	5,822,837	0.932	0.877–0.991	0.025	9,933,349	0.924	0.883–0.966	0.001
SIDS	9,933,349	1.018	0.916–1.132	0.735	5,822,837	0.990	0.808–1.213	0.924	9,933,349	1.018	0.916–1.132	0.735
Secondary outcomes												
Very low birth weight	9,933,349	1.010	0.978–1.042	0.558								
Early neonatal mortality	9,933,349	0.958	0.890–1.032	0.258								
Late neonatal mortality	9,911,272	0.863	0.793–0.940	0.001								
Post-neonatal mortality	9,904,292	0.954	0.900–1.010	0.106								
Infant mortality	9,933,349	0.937	0.904–0.971	<0.001								

Odds ratios indicate odds of developing outcome in period after versus period before July 2007, when smoke-free legislation was introduced. Primary models are adjusted for non-linear underlying time trends (via B-splines), month, maternal age, maternal marital status, sex, socioeconomic status, region, and urbanisation level and based on individual-level analysis of complete cases. Mortality models are furthermore adjusted for birth weight. For sensitivity analyses, model 1 is the complete case model with additional adjustment for parity, whereas in model 2 missing data for parity are imputed. OR = odds ratio; CI = confidence interval; SIDS = sudden infant death syndrome.
