# Supplementary material for: Impact of smoke-free legislation on perinatal and infant mortality: a national quasi-experimental study
Source: Sci Rep. 2015 Aug 13;5:13020. doi: 10.1038/srep13020 (PMC4534797; doi:10.1038/srep13020)
Supplement: Supplementary Information [file srep13020-s1.pdf]

Supplementary Information

Impact of smoke-free legislation on perinatal and infant mortality: a national quasi-experimental study

Jasper V Been, Daniel F Mackay, Christopher Millett, Jill P Pell, Onno CP van Schayck, Aziz Sheikh

Supplementary Table S1. Full multivariate logistic regression models for primary outcomes

	Low birth weight		Stillbirth		Neonatal mortality		SIDS	
	OR	95%CI	OR	95%CI	OR	95%CI	OR	95%CI
Smoke-free legislation	0.961	0.949-0.974	0.922	0.881-0.965	0.923	0.883-0.966	1.018	0.916-1.132
Month (ref = January)								
February	0.982	0.970-0.995	0.949	0.907-0.993	0.945	0.888-1.006	0.882	0.763-1.019
March	0.971	0.960-0.984	0.967	0.925-1.010	0.934	0.879-0.993	0.973	0.847-1.117
April	0.978	0.966-0.991	0.948	0.907-0.991	0.929	0.874-0.988	0.884	0.766-1.020
May	0.976	0.963-0.988	0.946	0.906-0.989	0.934	0.879-0.992	0.886	0.769-1.020
June	0.962	0.950-0.975	0.984	0.941-1.028	0.939	0.883-0.998	0.840	0.727-0.971
July	0.959	0.947-0.971	0.941	0.901-0.984	0.959	0.903-1.019	0.823	0.712-0.948
August	0.952	0.940-0.964	0.946	0.905-0.989	0.920	0.865-0.978	1.024	0.894-1.173
September	0.910	0.898-0.922	0.963	0.921-1.006	0.980	0.923-1.042	0.952	0.829-1.094
October	0.958	0.946-0.970	0.964	0.923-1.008	0.960	0.904-1.020	1.059	0.925-1.212
November	0.980	0.968-0.993	0.937	0.896-0.980	0.942	0.885-1.001	1.065	0.929-1.220
December	1.020	1.007-1.033	0.938	0.897-0.981	0.957	0.901-1.018	1.062	0.927-1.216
Maternal age (years; ref = 25-29)								
<20	1.261	1.248-1.275	1.010	0.972-1.049	1.136	1.080-1.195	2.216	2.020-2.430
20-24	1.132	1.123-1.140	0.994	0.967-1.022	1.075	1.035-1.116	1.551	1.429-1.683
30-34	0.928	0.921-0.935	1.028	1.002-1.054	0.971	0.938-1.005	0.844	0.768-0.927
35-39	1.023	1.014-1.032	1.110	1.078-1.143	1.014	0.973-1.057	0.846	0.752-0.951
≥40	1.246	1.227-1.265	1.421	1.356-1.489	1.130	1.054-1.122	0.839	0.672-1.049
Marital status (ref = not married)								
Married	0.794	0.790-0.799	1.036	1.015-1.057	1.072	1.043-1.102	0.403	0.376-0.431
Sex (ref = female)								
Male	0.853	0.848-0.857	1.123	1.103-1.144	1.331	1.298-1.365	1.430	1.348-1.517
Birth weight (grams; ref = 2500-3999)								
<1000	-	-	198.1	193.3-203.0	512.6	497.2-528.5	3.635	2.847-4.639
1000-1499	-	-	60.90	59.07-62.78	51.26	49.01-53.61	5.874	4.980-6.929
1500-2499	-	-	12.17	11.88-12.47	8.396	8.084-8.720	3.759	3.485-4.055
≥4000	-	-	0.916	0.874-0.960	0.778	0.725-0.834	0.502	0.437-0.573
IMD quintile (ref = 1 (most deprived))								
2	0.924	0.917-0.931	0.972	0.946-0.998	1.043	1.006-1.081	0.957	0.879-1.041
3	0.871	0.864-0.878	0.952	0.924-0.981	0.961	0.923-1.000	0.919	0.839-1.007
4	0.764	0.757-0.771	0.957	0.927-0.987	0.915	0.876-0.955	0.910	0.825-1.004
5 (least deprived)	0.762	0.754-0.770	0.932	0.898-0.967	0.884	0.840-0.931	0.841	0.749-0.943
Region (ref = Greater London)								
North East	0.901	0.889-0.913	0.992	0.946-1.039	1.093	1.023-1.168	1.156	1.004-1.331
North West	0.961	0.952-0.970	0.975	0.944-1.008	1.207	1.154-1.263	1.392	1.257-1.543
Yorkshire and the Humber	1.013	1.003-1.024	1.024	0.988-1.060	1.332	1.270-1.397	1.062	0.945-1.194
East Midlands	0.967	0.956-0.978	0.942	0.905-0.981	1.269	1.202-1.340	1.090	0.957-1.242
West Midlands	1.058	1.048-1.068	0.924	0.893-0.955	1.406	1.345-1.470	1.090	0.973-1.220
East of England	0.883	0.874-0.893	0.948	0.913-0.985	1.053	0.999-1.111	0.947	0.831-1.079
South East	0.841	0.830-0.851	0.941	0.902-0.981	0.948	0.892-1.009	1.099	0.959-1.260
South Central	0.873	0.862-0.883	0.949	0.910-0.990	1.054	0.993-1.118	1.058	0.919-1.220
South West	0.876	0.866-0.886	0.927	0.890-0.965	1.160	1.097-1.227	1.256	1.107-1.425
Urbanisation level (ref = rural)								
Urban	1.167	1.143-1.192	0.920	0.858-0.985	0.963	0.873-1.062	1.323	1.045-1.675

Odds ratios indicate odds of developing outcome in period after versus period before July 2007, when smoke-free legislation was introduced. Models are adjusted for non-linear underlying time trends (via B-splines), month, maternal age, maternal marital status, sex, socioeconomic status, region, and urbanisation level and based on

individual-level analysis of complete cases. Mortality models are furthermore adjusted for birth weight. Odds ratios are shown for smoke-free legislation and all covariates in the model (except the B-spline terms). Odds ratios for developing a distinct outcome are expressed relative to the reference group for each variable. SIDS = sudden infant death syndrome; OR = odds ratio; CI = confidence interval; IMD = Index of Multiple Deprivation.

Supplementary Table S2. Full multivariate logistic regression models for secondary outcomes

	Very low birth weight		Early neonatal mortality		Late neonatal mortality		Post-neonatal mortality		Infant mortality	
	OR	95%CI	OR	95%CI	OR	95%CI	OR	95%CI	OR	95%CI
Smoke-free legislation	1.010	0.978-1.042	0.958	0.890-1.032	0.863	0.793-0.940	0.954	0.900-1.010	0.937	0.904-0.971
Month (ref = January)										
February	1.004	0.972-1.036	0.960	0.894-1.030	0.918	0.816-1.033	0.928	0.856-1.007	0.937	0.891-0.985
March	1.014	0.983-1.046	0.940	0.877-1.007	0.932	0.832-1.044	1.016	0.940-1.098	0.963	0.917-1.011
April	1.044	1.012-1.077	0.940	0.877-1.007	0.911	0.812-1.023	0.994	0.919-1.076	0.953	0.907-1.001
May	1.028	0.997-1.061	0.947	0.884-1.015	0.915	0.816-1.026	0.992	0.917-1.072	0.953	0.908-1.001
June	0.988	0.957-1.020	0.968	0.903-1.038	0.885	0.787-0.995	0.959	0.885-1.038	0.943	0.897-0.990
July	0.972	0.942-1.002	0.967	0.903-1.035	0.959	0.856-1.075	0.987	0.913-1.067	0.967	0.921-1.015
August	0.962	0.932-0.992	0.940	0.877-1.007	0.894	0.797-1.005	1.092	1.012-1.179	0.982	0.936-1.031
September	0.933	0.904-0.962	0.987	0.921-1.057	0.987	0.880-1.106	1.019	0.943-1.102	0.993	0.946-1.043
October	0.992	0.962-1.024	0.992	0.926-1.062	0.902	0.804-1.013	1.082	1.002-1.168	1.004	0.957-1.054
November	0.996	0.965-1.028	0.928	0.865-0.995	1.002	0.895-1.123	1.075	0.995-1.162	0.991	0.944-1.042
December	1.008	0.977-1.040	0.936	0.873-1.004	1.040	0.929-1.164	1.046	0.968-1.131	0.990	0.943-1.040
Maternal age (years; ref = 25-29)										
<20	1.212	1.182-1.243	1.077	1.016-1.141	1.285	1.173-1.408	1.754	1.657-1.856	1.388	1.335-1.443
20-24	1.038	1.018-1.058	1.059	1.015-1.105	1.103	1.028-1.183	1.310	1.251-1.371	1.170	1.136-1.205
30-34	0.986	0.968-1.004	0.996	0.957-1.036	0.910	0.851-0.974	0.878	0.838-0.920	0.935	0.909-0.962
35-39	1.182	1.157-1.206	1.023	0.977-1.072	0.984	0.909-1.065	0.937	0.885-0.991	0.987	0.954-1.021
≥40	1.493	1.443-1.545	1.113	1.028-1.205	1.136	0.995-1.296	1.199	1.093-1.316	1.167	1.102-1.236
Marital status (ref = not married)										
Married	0.728	0.718-0.738	1.092	1.059-1.127	1.001	0.950-1.054	0.859	0.830-0.890	0.985	0.964-1.007
Sex (ref = female)										
Male	0.993	0.981-1.006	1.324	1.287-1.363	1.324	1.262-1.389	1.342	1.300-1.386	1.340	1.313-1.368
Birth weight (grams; ref = 2500-3999)										
<1000	-	-	589.2	568.9-610.2	261.2	246.6-276.8	82.99	79.04-87.13	308.8	301.0-316.7
1000-1499	-	-	55.82	52.96-58.84	40.63	37.37-44.18	16.72	15.63-17.88	33.54	32.32-33.81
1500-2499	-	-	9.134	8.732-9.555	6.881	6.416-7.379	5.310	5.090-5.539	6.808	6.619-7.002
≥4000	-	-	0.887	0.818-0.961	0.558	0.484-0.643	0.532	0.490-0.577	0.649	0.615-0.684
IMD quintile (ref = 1 (most deprived))										

2	0.905	0.889-0.922	1.054	1.012-1.097	1.010	0.944-1.080	1.016	0.971-1.063	1.032	1.003-1.063
3	0.849	0.832-0.866	0.959	0.916-1.003	0.983	0.911-1.060	0.914	0.868-0.962	0.938	0.908-0.969
4	0.700	0.685-0.715	0.932	0.887-0.979	0.894	0.824-0.971	0.887	0.841-0.937	0.898	0.868-0.930
5 (least deprived)	0.677	0.660-0.695	0.894	0.843-0.948	0.878	0.796-0.969	0.805	0.754-0.860	0.847	0.813-0.882
Region (ref = Greater London)										
North East	0.806	0.780-0.833	1.105	1.025-1.192	1.055	0.935-1.190	0.940	0.867-1.020	1.030	0.977-1.085
North West	0.840	0.821-0.859	1.237	1.175-1.303	1.133	1.042-1.232	1.124	1.063-1.188	1.176	1.134-1.219
Yorkshire and the Humber	0.890	0.867-0.912	1.356	1.283-1.432	1.254	1.149-1.370	1.153	1.087-1.223	1.260	1.214-1.310
East Midlands	0.855	0.832-0.879	1.334	1.254-1.419	1.107	0.999-1.227	0.953	0.887-1.023	1.135	1.086-1.186
West Midlands	0.972	0.951-0.995	1.543	1.468-1.622	1.072	0.982-1.170	1.027	0.969-1.089	1.248	1.204-1.294
East of England	0.816	0.795-0.838	1.115	1.049-1.185	0.915	0.826-1.014	0.933	0.872-0.998	1.001	0.959-1.045
South East	0.740	0.718-0.762	0.997	0.929-1.069	0.844	0.750-0.949	0.850	0.787-0.919	0.905	0.862-0.951
South Central	0.826	0.802-0.850	1.070	1.000-1.145	1.019	0.913-1.137	0.940	0.872-1.013	1.007	0.960-1.056
South West	0.813	0.791-0.836	1.217	1.141-1.297	1.022	0.919-1.138	0.954	0.888-1.025	1.073	1.026-1.123
Urbanisation level (ref = rural)										
Urban	1.180	1.120-1.244	0.901	0.807-1.006	1.149	0.937-1.409	1.161	1.017-1.326	1.040	0.960-1.127

Odds ratios indicate odds of developing outcome in period after versus period before July 2007, when smoke-free legislation was introduced. Models are adjusted for non-linear underlying time trends (via B-splines), month, maternal age, maternal marital status, sex, socioeconomic status, region, and urbanisation level and based on individual-level analysis of complete cases. Mortality models are furthermore adjusted for birth weight. Odds ratios are shown for smoke-free legislation and all covariates in the model (except the B-spline terms). Odds ratios for developing a distinct outcome are expressed relative to the reference group for each variable. OR = odds ratio; CI = confidence interval; IMD = Index of Multiple Deprivation.

Supplementary Table S3. Impact of smoke-free legislation on mortality outcomes with and without adjustment for birth weight

	Primary analysis (adjusted for birth weight)				Sensitivity analysis (not adjusted for birth weight)			
Primary outcomes	N	OR	95%CI	P-value	N	OR	95%CI	P-value
Stillbirth	9,984,278	0.922	0.881-0.965	<0.001	10,026,641	0.934	0.895-0.975	0.002
Neonatal mortality	9,933,349	0.924	0.883-0.966	0.001	9,975,300	0.956	0.919-0.995	0.028
SIDS	9,933,349	1.018	0.916-1.132	0.735	9,975,300	0.998	0.898-1.109	0.970
Secondary outcomes								
Early neonatal mortality	9,933,349	0.958	0.890-1.032	0.258	9,975,300	0.999	0.936-1.068	0.988
Late neonatal mortality	9,911,272	0.863	0.793-0.940	0.001	9,952,103	0.895	0.824-0.972	0.008
Post-neonatal mortality	9,904,292	0.954	0.900-1.010	0.106	9,944,960	0.961	0.909-1.017	0.173
Infant mortality	9,933,349	0.937	0.904-0.971	<0.001	9,975,300	0.958	0.927-0.990	0.011

Odds ratios indicate odds of developing outcome in period after versus period before July 2007, when smoke-free legislation was introduced. Primary models are adjusted for non-linear underlying time trends (via B-splines), month, maternal age, maternal marital status, sex, birth weight, socioeconomic status, region, and urbanisation level and based on individual-level analysis of complete cases. Sensitivity analyses include all these variables except birth weight. OR = odds ratio; CI = confidence interval; SIDS = sudden infant death syndrome

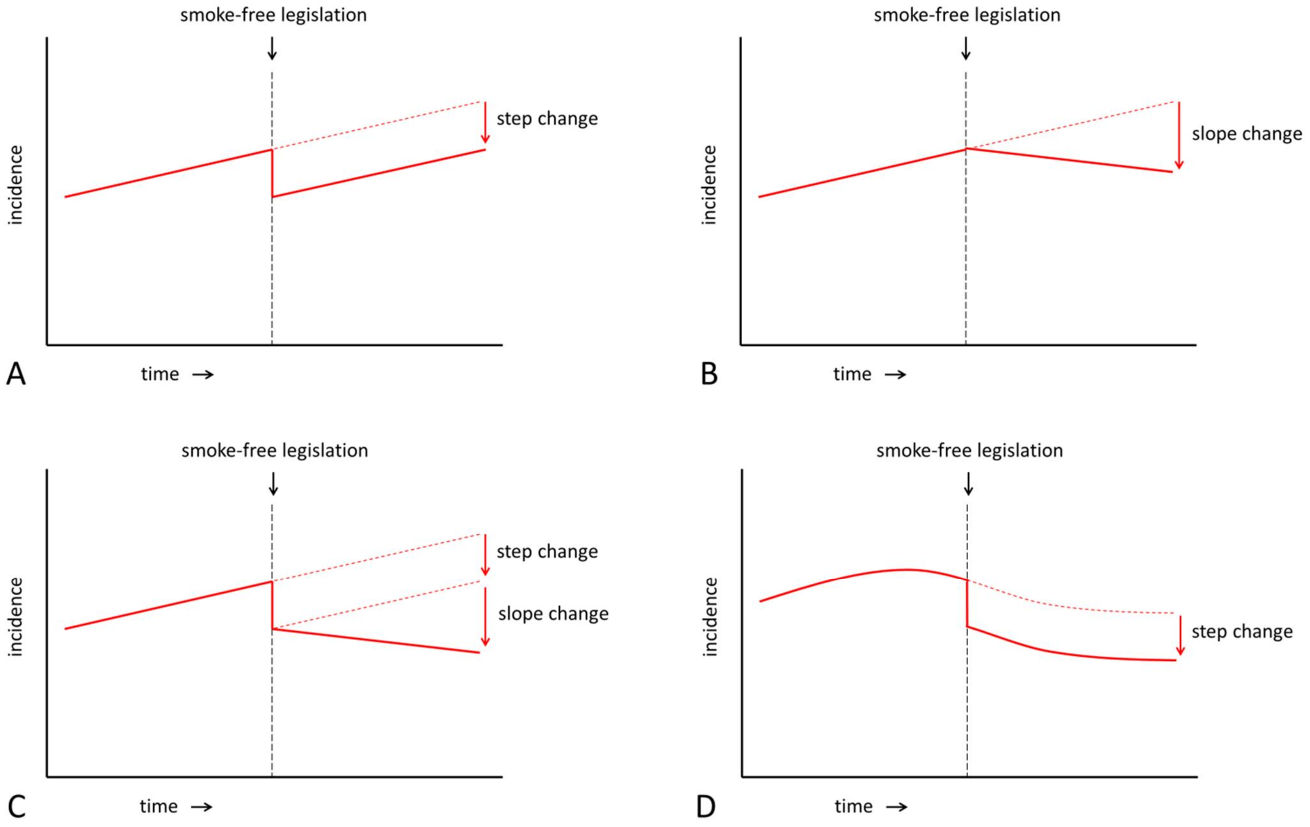

Supplementary Figure S1. Simplified graphical model representations. The different models considered for modelling the impact on smoke-free legislation on each outcome are displayed. Hypothetical temporal incidence trends and effect sizes are shown. A: linear time trend model with sudden ('step') change in incidence; B: linear time trend model with gradual ('slope') change in incidence; C: linear time trend model with step and slope change; D: non-linear time trend model with step change in incidence (three such models where evaluate with linear, quadratic, and cubic B-splines, respectively).

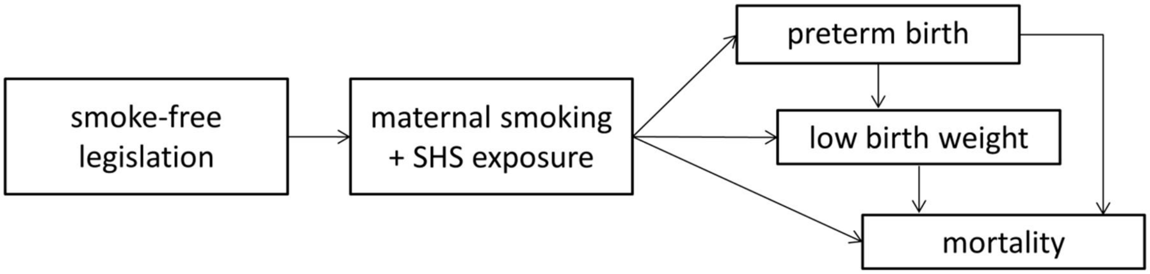

Supplementary Figure S2. Theoretical framework of causal pathways between smoke-free legislation and perinatal outcomes. Directed acyclic graph indicating likely causal pathways (all negative associations) based on previous studies. Note how preterm birth is an intermediate variable on the causal pathway from smoke-free legislation to reductions in low birth weight and perinatal mortality.

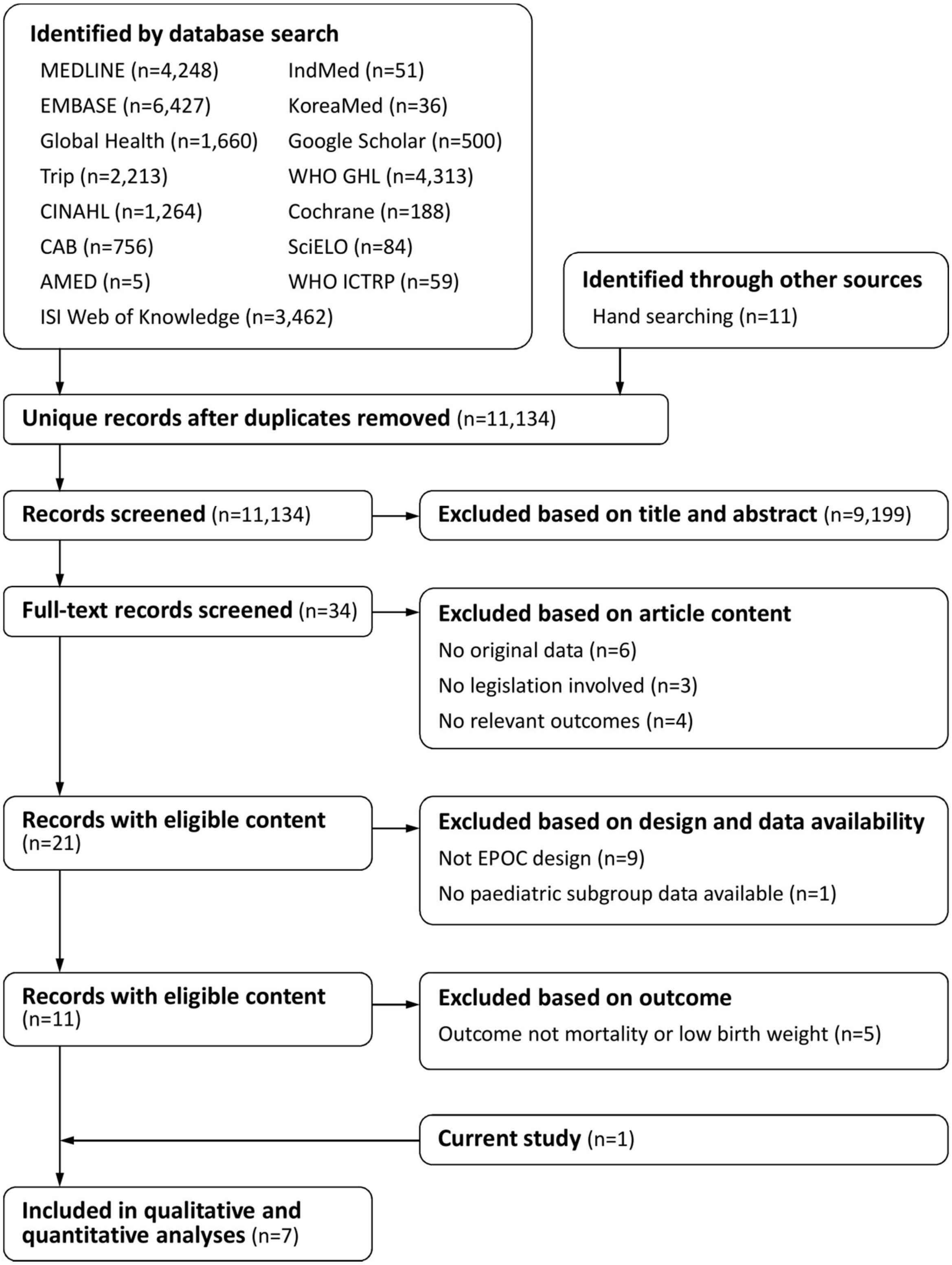

Supplementary Figure S3. Updated systematic review: study selection. Searches were performed in April 2014. Inclusion was restricted to studies of mortality and perinatal outcomes. EPOC=Cochrane Effective Practice and Organization of Care.
